# METABOLIC SYNDROME AND LATE-LIFE DEPRESSION AMONG MEXICAN AMERICANS: THE ROLE OF NATIVITY

**DOI:** 10.1093/geroni/igz038.1168

**Published:** 2019-11-08

**Authors:** Weihui Zhang

**Affiliations:** University at Albany, Rensselaer, United States

## Abstract

Metabolic syndrome (MS) has been reported to predict depression. However, studies evaluating if there are differences by nativity status among Mexican Americans are scarce. This study aims to examine the association between metabolic syndrome and depression among Mexican-American older adults. We also evaluated the role of nativity, sociodemographic and health risk factors. We use three waves (2006-2013) from the Hispanic Established Populations for the Epidemiologic Study of the Elderly (HEPESE; N=1,542, mean age =83.45 in 2006). MS was defined according to the National Cholesterol Education Programme (NCEP-ATP III) using abdominal obesity, use of antihypertensive medication, and insulin. Depression was ascertained by self-report of a CES-D score greater than 16. We applied random-effect logistic regression models which accounted for inter-individual correlation and adjusted for age, sex, education, smoking, alcohol use, physical performance, and self-esteem. We also tested for interaction between MS and nativity. Approximately 30% of foreign-born and 22% of US-born reported depression. The prevalence of MS was higher in the Foreign-born when compare to the US-born (5.89% vs. 5.35%). In the total sample, MS was associated with a higher risk of depression (OR=4.34, p=0.007). Foreign-born Mexican Americans were more likely to have depression (OR=1.70, p=0.002) when compared to US-born; however, foreign-born with MS reported lower depression (OR=0.26, p=0.052) after adjusting for potential confounders. Our finding adds to the concept of “metabolic depression,” and further highlights the importance of evaluating nativity to explain the differences in physical and psychological health among a sample of the Hispanic population at old age.

